# Association between DAZL polymorphisms and susceptibility to male infertility: systematic review with meta-analysis and trial sequential analysis

**DOI:** 10.1038/srep04642

**Published:** 2014-04-10

**Authors:** Simin Zhang, Qiuqin Tang, Wei Wu, Beilei Yuan, Chuncheng Lu, Yankai Xia, Hongjuan Ding, Lingqing Hu, Daozhen Chen, Jiahao Sha, Xinru Wang

**Affiliations:** 1 State Key Laboratory of Reproductive Medicine, Institute of Toxicology, Nanjing Medical University, Nanjing 211166, China; 2 Key Laboratory of Modern Toxicology of Ministry of Education, School of Public Health, Nanjing Medical University, Nanjing 211166, China; 3 Department of Epidemiology and Biostatistics, School of Public Health, Nanjing Medical University, Nanjing 211166, China; 4 State Key Laboratory of Reproductive Medicine, Department of Obstetrics, Nanjing Maternity and Child Health Care Hospital Affiliated to Nanjing Medical University, Nanjing 210004, China; 5 State Key Laboratory of Reproductive Medicine, Wuxi Maternal and Child Health Care Hospital Affiliated to Nanjing Medical University, Wuxi 214002, China; 6 These authors contributed equally to this work.

## Abstract

Several studies have investigated the association between polymorphisms in the Deleted in AZoospermia-Like (*DAZL*) gene and male infertility risk, but with inconsistent results. We aimed to derive a more precise estimation of the relationship, therefore a meta-analysis was performed. A total of 13 case-control studies, including 2556 cases and 1997 controls, were selected. Two polymorphisms in *DAZL* were investigated, namely *T12A* (Thr12 → Ala) and *T54A* (Thr54 → Ala). Our meta-analysis showed that A > G is a risk factor for male infertility (*P* = 0.047, OR = 1.262, 95%CI = 1.003–1.587). However, when using trial sequential analysis (TSA) to confirm, we found that A > G risk effect turned out to be false positive. In addition, significant association was found between the *T54A* polymorphism and male infertility under co-dominant model (AG vs. AA: OR = 4.364, 95%CI = 2.207–8.630, *P* < 0.001) and dominant model (OR = 4.584, 95%CI = 2.320–9.058, *P* < 0.001). Stratified analysis showed that significantly strong association between *T54A* polymorphism and male infertility was present only in Asians, but not in Caucasians. Further studies of *T12A* and *T54A* with their biological functions are needed to understand the role of these polymorphisms in the development of male infertility.

Globally, around 15% of heterosexual couples suffer from inability to conceive their own child without any assistance^1^. In half of the cases, males are the ones to blame. Many reasons can lead to male infertility, such as malformations of the reproductive tract^2^, infection^3^ and chemical exposures^4^. Still, 50–70% of male infertility is of unknown etiology and that much of this is likely genetic. To unveil the reason, intensive research for genetic causes of male infertility has been performed in recent years.

*DAZL* (deleted in azoospermia-like) is an autosomal homologue of the *DAZ* (deleted in azoospermia), a gene cluster which gets deletions in at least 10% of males with azoospermia or oligozoospermia^5^,^6^,^7^,^8^. As a result, *DAZL* has always been seen as a promising candidate for male infertility. Although varies in detail, studies aiming at *DAZL* expression share the same point: DAZL plays an important role in the human spermatogenic processes and might function as a translational activator through regulating mRNA expression^9^,^10^,^11^,^12^, though its mechanism is still largely unknown. In a study on human beings, DAZL protein is shown to be present in male germ cells in many stages during spermatogenesis^6^, and the nuclear localization of DAZL protein is also observed in gonocytes and spermatogonia. Another study has demonstrated that DAZL protein is located in the nuclei of gonocytes, and relocalized to the cytoplasm in adults^13^. There is ever-growing evidence on animals that confirms similar points: in two experiments performed on transgenic mice with a *Dazl* null background, either carrying human *DAZL* or human *DAZ* shows a partial rescue of the *Dazl* knockout mice. Although the mice remain infertile, both transgenes enable prophase spermatocytes to be produced^14^.

Recently, a number of molecular epidemiological studies have been conducted to examine the association between *DAZL* polymorphisms and male infertility in diverse populations. Among them, two non-synonymous single nucleotide polymorphisms (SNPs) at nucleotide position 260 (exon 2) and 386 (exon 3), resulted by the amino acid exchange *T12A* (Thr12 → Ala) and *T54A* (Thr54 → Ala) respectively, are most frequently studied^15^,^16^,^17^,^18^,^19^,^20^,^21^,^22^,^23^,^24^,^25^,^26^,^27^. However, the results of these studies are inconsistent or even contradictory. Most studies till date have analyzed these polymorphisms in rather small sample size, leading to under-estimation of the association. To estimate the effect of polymorphisms and risk of male infertility, as well as to quantify the potential between-study heterogeneity, we conducted a meta-analysis on 13 eligible and published case-control studies.

## Results

### Study characteristics

**Figure 1:**
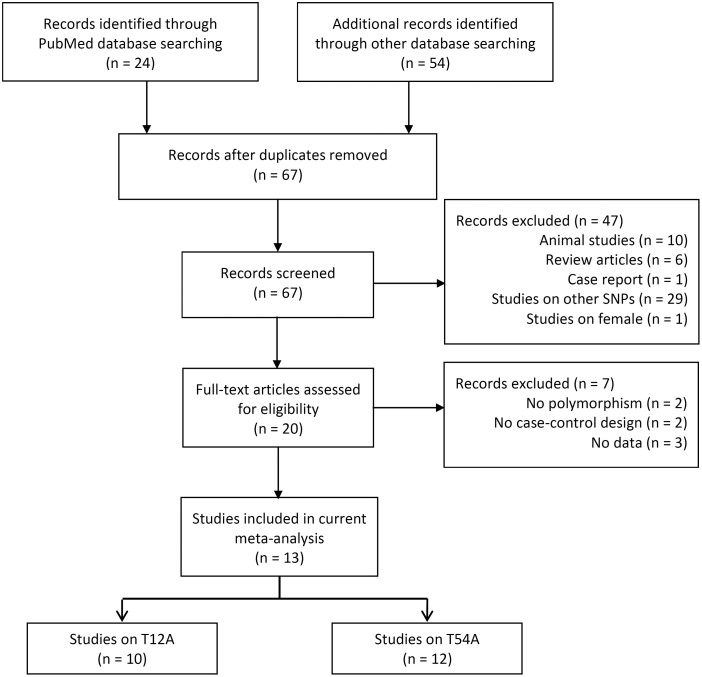
Flow diagram of the study selection process.

Through the literature search and selection based on inclusion criteria, 13 articles were identified by reviewing potentially relevant articles (Figure 1). The characteristics of the selected studies are shown in Table 1 and Table 2.

*T12A* polymorphism: A total of 10 studies were included in the meta-analysis with 2174 cases and 1594 controls. The number of cases included in the studies varied from 92 to 660, with a mean (± SD) of 217.40 (±163.16), and the number of controls varied from 40 to 350, with a mean (± SD) of 159.40 (±89.67).

*T54A* polymorphism: In total, twelve studies met the inclusion criteria and were selected for the meta-analysis including 2456 cases and 1897 controls. The number of cases included in the studies varied from 71 to 660, with a mean (± SD) of 204.67 (±151.70), and the number of controls varied from 40 to 350, with a mean (± SD) of 158.08 (±85.35).

### Meta-analysis of T12A polymorphism and male infertility

**Figure 2:**
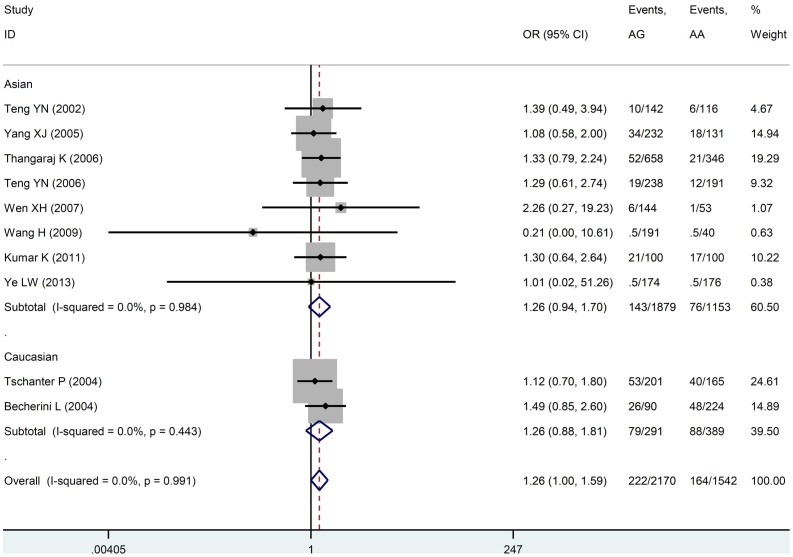
Forest plot of the *T12A* polymorphism and male infertility risk in the co-dominant model. Studies are plotted according to the last name of the first author and followed by the publication year in parentheses. Horizontal lines represent 95% CI. Each square represents the OR point estimate and its size is proportional to the weight of the study. The diamond (and broken line) represents the overall summary estimate, with confidence interval given by its width. The unbroken vertical line is at the null value (OR = 1.0). CI, confidence interval; OR, odds ratio.

The evaluation of the association between *T12A* polymorphism and male infertility risk is summarized in Table 3, Figure 2 and Figure S1. No significant association was observed between *T12A* polymorphism and male infertility under dominant model and recessive model (for AG + GG vs. AA: OR = 1.244, 95%CI = 0.994–1.557, *P* = 0.057; for GG vs. AA + AG: OR = 0.840, 95%CI = 0.384–1.834, *P* = 0.661). However, we found that when companied with AA genotype, AG is significantly associated with male infertility (*P* = 0.047, OR = 1.262, 95%CI = 1.003–1.587). Furthermore, in the subgroup analyses based on ethnicity and case types, no significant association was found between the *T12A* polymorphism and male infertility in the Caucasian group (*P* = 0.213, OR = 1.259, 95%CI = 0.876–1.808), Asian group (*P* = 0.122, OR = 1.264, 95%CI = 0.939–1.700), azoospermia group (*P* = 0.102, OR = 1.411, 95%CI = 0.934–2.129) and OAT group (*P* = 0.986, OR = 0.986, 95%CI = 0.616–1.579).

### Meta-analysis of T54A polymorphism and male infertility

**Figure 3:**
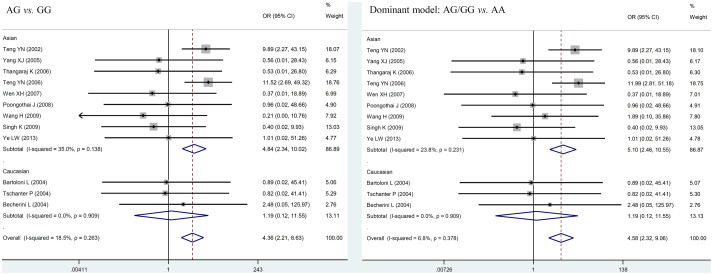
Forest plot of the *T54A* polymorphism and male infertility risk in the co-dominant model and dominant model. Studies are plotted according to the last name of the first author and followed by the publication year in parentheses. Horizontal lines represent 95% CI. Each square represents the OR point estimate and its size is proportional to the weight of the study. The diamond (and broken line) represents the overall summary estimate, with confidence interval given by its width. The unbroken vertical line is at the null value (OR = 1.0). CI, confidence interval; OR, odds ratio.

The evaluation of the association between *T54A* polymorphism and male infertility risk is summarized in Table 4 and Figure 3. In the overall analysis, significant association was found between *T54A* polymorphism and male infertility under co-dominant model (for AG vs. AA: OR = 4.364, 95%CI = 2.207–8.630, *P* < 0.001) and dominant model (OR = 4.584, 95%CI = 2.320–9.058, *P* < 0.001). To clarify the potential ethnic difference, subgroup analysis by ethnicity of study population was also conducted. Significant association was found between T54A polymorphism and the risk of male infertility in the subgroups of Asians under co-dominant model (for AG vs. AA: OR = 4.842, 95%CI = 2.339–10.025, *P* < 0.001) and dominant model (OR = 5.097, 95%CI = 2.463–10.546, *P* < 0.001). While no such conclusion can be found in Caucasian group under co-dominant model (for AG vs. AA: OR = 1.195, 95%CI = 0.124–11.549, *P* = 0.878) and dominant model (OR = 1.195, 95%CI = 0.124–11.549, *P* = 0.878).

### Publication Bias and Small-study Effects

Begg's funnel plot and Egger's test were performed to assess the publication bias of literatures. For *T12A*, the shape of the funnel plot did not reveal any evidence of obvious asymmetry (Figure S2). Moreover, the Egger's test was used to provide statistical evidence of funnel plot symmetry. The results did not suggest any evidence of publication bias or small-study effects (*P* = 0.678 for AG vs. AA; *P* = 0.388 for GG vs. AA; *P* = 0.308 under dominant model; and *P* = 0.356 under recessive model). For *T54A*, Egger's test revealed the existence of significant publication bias and small-study effects (*P* < 0.001 for AG vs. AA; *P* = 0.021 for GG vs. AA; *P* < 0.001 under dominant model; and *P* = 0.022 under recessive model).

### Sensitive analysis

Sensitivity analyses were conducted to determine whether modification of the inclusion criteria of the meta-analysis affected the final results. We conducted the sensitive analyses on *T12A* under co-dominant model by omitting one study at a time in the calculation of the summary outcome. The results showed that no study fundamentally changed the relationship between *T12A* and risk of male infertility. As for *T54A*, although the sample size for cases and controls in all eligible studies varies, the corresponding pooled ORs were not qualitatively altered with or without any study (Figure S3).

### Trial sequential analysis

**Figure 4:**
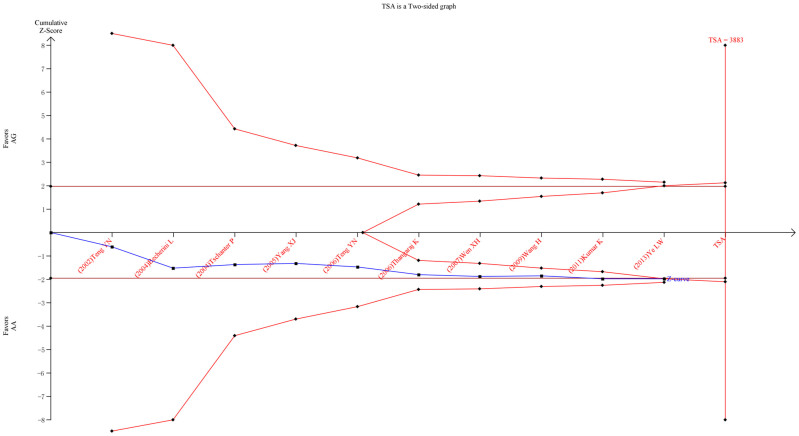
Trial sequential analysis of the *T12A* polymorphism and male infertility risk in the co-dominant model. The diversity-adjusted required information size (3883 participants) was based on a relative risk reduction of 10%; an alpha of 5% and a beta of 5%. The blue line represents the cumulative Z-score of the meta-analysis. The red straight represent the conventional *P* = 0.05 statistical boundaries. The inward sloping red lines represent the truncated trial sequential monitoring boundaries.

**Figure 5:**
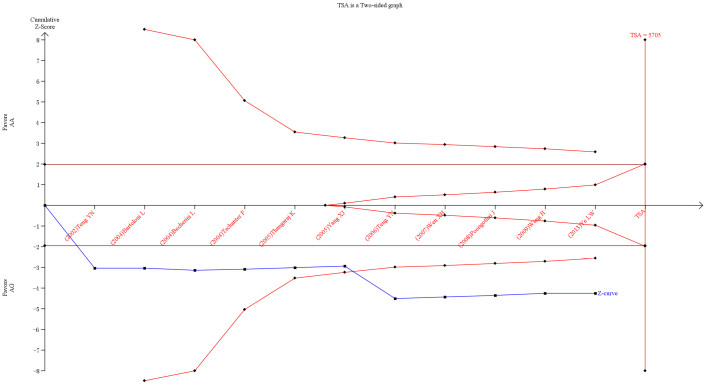
Trial sequential analysis of the *T54A* polymorphism and male infertility risk in the co-dominant model. The diversity-adjusted required information size (5705 participants) was based on a relative risk reduction of 10%; an alpha of 5% and a beta of 5%. The blue line represents the cumulative Z-score of the meta-analysis. The red straight represent the conventional *P* = 0.05 statistical boundaries. The inward sloping red lines represent the truncated trial sequential monitoring boundaries.

With the settings mentioned in the Methods section, we calculated the required information size to 3883 patients. As the number of patients included in the meta-analysis did not exceed the required information size, we also applied futility boundaries to potentially facilitate a firm ‘negative’ conclusion. The resulting trial sequential analysis is shown in Figure 4. The trial sequential analysis showed that the cumulative Z-curve (blue line) did cross the conventional *P* = 0.05 boundary (red straight lines), but failed to cross the trial sequential monitoring boundaries for harm (inward sloping red lines). Neither did the cumulative Z-curve reach the trial sequential monitoring boundaries for futility. Within the set assumptions for confidence and effect size, we found that in *T12A* the A > G risk effect turned out to be false positive. As for *T54A*, the positive result was confirmed (Figure 5).

## Discussion

Spermatogenesis is a complex process of mitotic and meiotic divisions of germ cells finally resulting in the formation of haploid spermatozoa^28^. A highly coordinated expression of genes and a subtle post-transcriptional regulation are therefore, crucial for normal germ cell development^29^. A most recent meta-analysis on *T12A* polymorphism included six studies^30^. Although the previous meta-analysis may be involved some parts of the relationship between *T12A* polymorphism and male infertility risk, its eligible studies are not quite comprehensive. The lack of four published studies^17^,^24^,^25^,^26^ may lead to the decrease of studies and may cause a deviation to the final result. Also it failed to involve explore the ethnicity background differences between studies. In our present study, we included 10 studies, dating back from 2002 to 2014. Moreover instead of comparing AA vs. GG/AG, we compared the distribution of AG vs. AA, GG vs. AA, AG/GG vs. AA (dominant model) and GG vs. AA/AG (recessive model) among case group and control group. Interestingly, we found that when companied with AA genotype, AG is a risk factor to male infertility (*P* = 0.047, OR = 1.262, 95%CI = 1.003–1.587). However, when using trial sequential analysis (TSA) to confirm, we found that A > G risk effect turned out to be false positive. Our analysis suggests that further exploration of the true association between *DAZL* polymorphism and male infertility is demanded.

The literature on the relationship between *T54A* polymorphism and male infertility risk is replete with small studies that report controversial findings. No clear consensus has been reached and there is no meta-analysis. Only in Taiwanese population *T54A* polymorphism do pose a significant difference between case and control group. We have the following assumptions: firstly, the studies from mainland China with a relatively small sample size (average of 171 cases and 89 controls) might lack of adequate power to draw a fair conclusion. Inconsistent associations in Caucasian data indicate that there may be differences in the magnitude of the contribution to male infertility susceptibility by ethnicity. Secondly, the progress of male infertility has long been seen as the outcome of the interaction between gene and environment^31^. Take the recent mice experiment as an example, DAZL was observed to be translocated to stress granules (SGs) upon heat stress. Furthermore, SG assembly activity was significantly diminished in the early male germ cells of *Dazl*-knockout mice. The findings suggest DAZL's interactions with environment is essential in protecting male germ cells from heat stress^32^. Taiwan is geographically far away from China mainland, this discrepancy may also be attributed to the different climate, diet, lifestyle and economic status. Hence, chances are high that the conflicting results of genetic studies of *DAZL* can be explained when taking interaction into account. Thirdly, apart from interactions, SNPs' joint effects and *DAZL* haplotypes should also be considered. An individual with a clinical disorder is not the product of the single gene that is disrupted, but that the genetic disruption is embedded within the context of that individual's entire genome^33^. In a study carried in Taiwan, protective haplotypes occurred in 72% of fertile controls and deleterious haplotypes occurred in 59.2% of infertile men, suggesting the association between autosomal *DAZL* haplotypes and human spermatogenic failure^23^,^34^,^35^. Last but not the least, novel missense mutations in *DAZL* and DAZL's role of epigenetic mechanisms in male infertility should be taken into future studies^36^. Teng *et al.*^37^ found that 792G > A was more prevalent in the infertile men and it affected sperm concentration and motility. In a recent study, abnormal DNA methylation of the *DAZL* promoter is found to be closely associated with oligozoospermia^38^.

**Figure 6:**
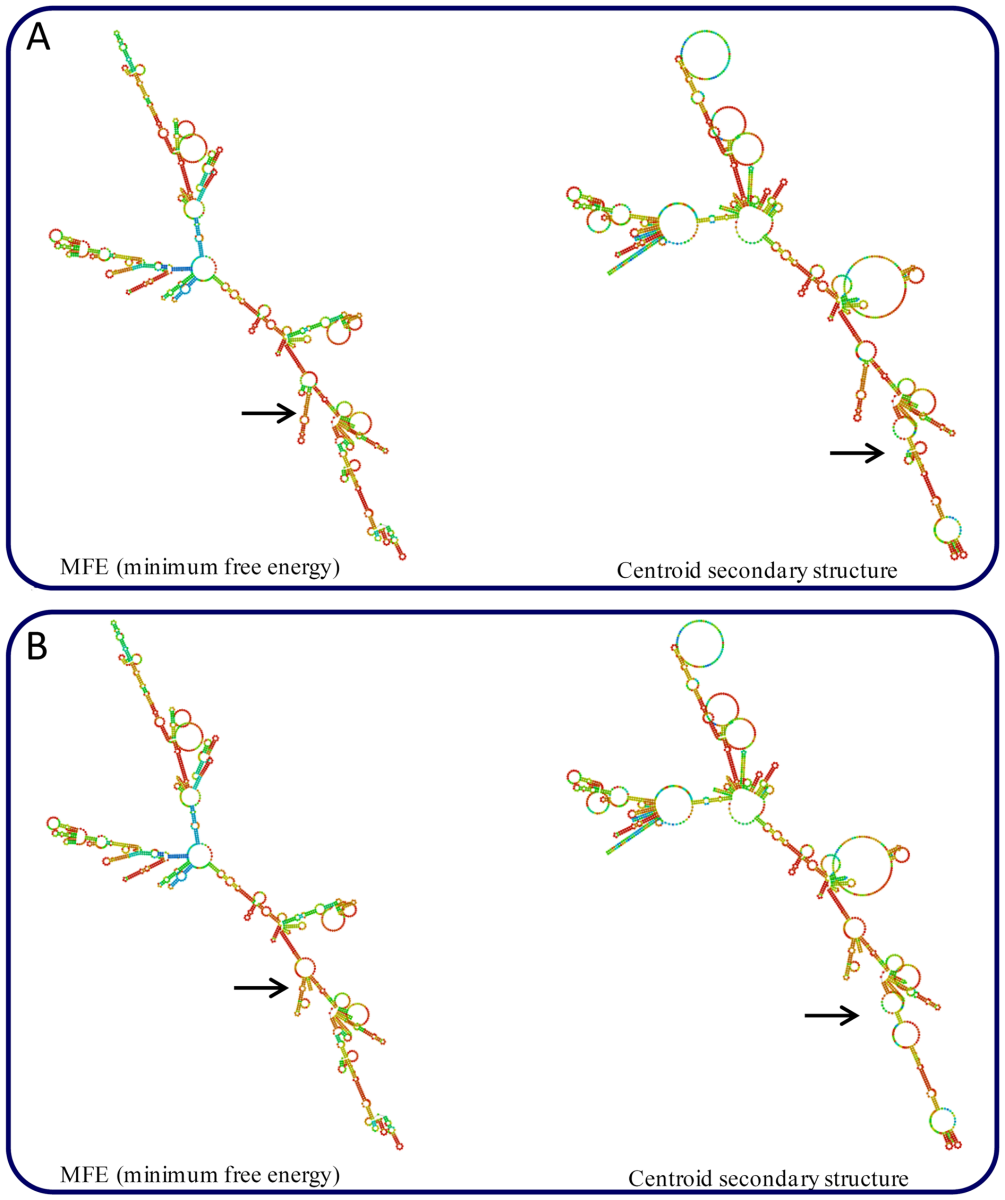
Prediction of the secondary structure of mRNA sequence containing the *T54A* variants. All structures were predicted with RNAfold software (http://rna.tbi.univie.ac.at/cgi-bin/RNAfold.cgi).

Our meta-analysis suggests that *DAZL* has a relationship with male infertility, but the exact molecular mechanisms of how the variant *T54A* (located on exon 3) affects male infertility is unknown. In order to explore the probable mechanisms, a secondary structure of the *DAZL* mRNA sequence prediction was performed, using RNAfold (http://rna.tbi.univie.ac.at/cgi-bin/.RNAfold.cgi)^39^. Pointed areas in Figure 6 show significant changes of RNA structure both under MFE (minimum free energy) model and centroid secondary structure, suggesting the *T54A* polymorphism might affect the stability of the RNA or the interaction of the RNA with other macromolecules.

However, some limitations need to be addressed in our meta-analysis. Firstly, some studies with small sample size may not have enough statistical power to explore the real association and are thought to be more likely to report larger beneficial effects than large trials^40^. Secondly, our results were based on unadjusted estimates, while a more precise analysis should be conducted if all individual data were available, which would allow for the adjustment by other co-variants and even novel algorithms to limit the wide-spreading data fitting problems^41^. Thirdly, inclusion of zero-event trials can sometimes decrease the effect size estimate and narrow confidence intervals^42^.

In conclusion, our meta-analysis suggested that further exploration of the true association between *T12A* polymorphism and male infertility is demanded. At the same time, significant associations were found between the *T54A* polymorphism and male infertility under co-dominant model and dominant model. Concerning male infertility with multifactorial etiology, more studies with a large sample size and stratified by different ethnic background, environmental exposure or other risk factors are needed to be performed to clarify the possible roles of *DAZL* polymorphism in the pathogenesis of male infertility in the future.

## Methods

### Study selection

We systematically collected published studies from 2002 to 2014 by searching both the common English database (PubMed) and the Chinese literature databases [CNKI (http://www.cnki.net), VIP (http://www.cqvip.com), and WanFang (http://www.wanfangdata.com.cn)]. The following searching phrases were used: (*DAZL* or deleted-in-azoospermia-like) and (polymorphism or polymorphisms) and male infertility. Data from single reports were extracted and summarized in Table 1 and Table 2.

Two independent reviewers assessed the full text of eligible studies through the above databases. Additional studies were identified by a manually search of references of original or review articles on this topic. The inclusion criteria were: (i) evaluation of *T12A*, *T54A* polymorphism and male infertility risk; (ii) studied on human beings; (iii) case-control study design; (iv) had detailed genotype frequency of cases and controls or could be derived from the article text; and (v) the full text of the paper could be obtained.

### Data extraction and verification

Firstly, two reviewers independently screened the citations using the inclusion criteria. Next, one reviewer extracted the data and the other cross-checked the data. Any disagreement was resolved by reviewing and discussing. The main elements of the extracted information included the first author's name, year of publication, country or region of origin, ethnicity, number of cases and controls. Different ethnicity was categorized as Asian and Caucasian.

### Quality score assessment

The quality of the studies was evaluated using the Newcastle–Ottawa scale (NOS)^43^. The NOS ranges between zero (worst) and nine stars (best). Each study was assessed based on three broad perspectives: selection, comparability, and exposure (Table S1). The ultimate score of six stars or more was regarded as high-quality.

### Statistical analysis

All statistical analyses were carried out using STATA 12.0 (STATA Corp, LP) and *P* < 0.05 was considered to be significant. The strength of association between the polymorphisms and male infertility risk was assessed by ORs with 95% CIs. The combined ORs were respectively calculated for three genetic models (co-dominant model, dominant model and recessive model). Furthermore, we conducted subgroup analyses by stratifying ethnicity into Caucasians and Asians separately and case types [oligoasthenoteratozoospermia (OAT), azoospermia]. Taking consideration of possible between-study heterogeneity, a statistical test for heterogeneity was performed. If the *P* value for heterogeneity was >0.10 and *I*^*2*^ < 50%, indicating an absence of heterogeneity between studies, and we used the fixed-effect model to evaluate the summary OR. In contrast, if the *P* value for heterogeneity was ≤0.10 or *I*^2^ ≥ 50%, indicating a high extent of heterogeneity between studies, and we used the random-effect model to evaluate the summary OR. A fixed-effect model using the Mantel-Haenszel method and a random-effects model using the DerSimonian and Laird method were used to combine values from studies. Begg's and Egger's test and funnel plots were utilized to provide a diagnosis of publication bias and small-study effects (linear regression asymmetry test). Due to the lack of polymorphism, several trials report zero events in both infertility and control groups. Exclusion of these trials could inflate the size of pooled treatment. To compensate for this we applied a continuity correction of 0.5 in zero-event trials^44^. Furthermore, we conducted a sensitive analysis to investigate the influence of a single study on the overall effect estimate by omitting one study in each turn.

### Trial sequential analysis

A novel statistical analysis software, TSA (The Copenhagen Trial Unit, Center for Clinical Intervention Research, Denmark), can adjust the threshold for statistical significance according to the quantified strength of evidence and the impact of multiplicity. A meta-analysis may result in type I errors and type II errors if data are sparse or if there is repeated testing for significance when new trials are added^45^,^46^.

To minimize the risk of type-I errors, TSA program was used. TSA combines conventional meta-analysis methodology with meta-analytic sample size considerations (i.e., required information size) and methods for repeated significance testing on accumulating data in trials. Recent studies show that TSA has the potential to make conclusions more reliable than those traditional meta-analyses^45^,^47^. The required information size was calculated according to an overall type-I error of 5%, a power of 95% and a relative risk reduction (RRR) assumption of 10%. A continuity correction of 0.5 was also applied in zero-event trials.

## Supplementary Material

Supplementary Information supplementary information

## Figures and Tables

**Table 1:** Main characteristics of all studies of *T12A* genotypes included in the meta-analysis

							Case			Control		
First author	Year	Country or Region	Ethnicity	Case	Control	Group	AA	AG	GG	AA	AG	GG
Teng YN^20^	2002	Taiwan China	Asian	142	160	Total	132	10	0	110	6	0
Tschanter P^16^	2004	Germany	Caucasian	202	165	Total	148	53	1	125	40	0
Becherini L^22^	2004	Italy	Caucasian	92	229	Total	64	26	2	176	48	5
Yang XJ^18^	2005	Japan	Asian	234	131	Total	198	34	2	113	18	0
						OAT^a^	85	7	1			
						Azoospermia	113	27	1			
Thangaraj K^19^	2006	India	Asian	660	350	Total	606	52	2	325	21	4
						OAT^a^	58	4	0			
						Azoospermia	548	48	2			
Teng YN^23^	2006	Taiwan China	Asian	231	191	Total	219	19	0	179	12	0
Wen XH^17^	2007	Mainland China	Asian	144	53	Total	138	6	0	52	1	0
						OAT^a^	112	5	0			
Wang H^26^	2009	Mainland China	Asian	196	40	Total	190	0	6	39	0	1
Kumar K^24^	2011	India	Asian	100	100	Total	79	21	0	83	17	0
						OAT^a^	79	21	0			
Ye LW^25^	2013	Mainland China	Asian	173	175	Total	173	0	0	175	0	0
						OAT^a^	173	0	0			

OAT: oligoasthenoteratozoospermia.

**Table 2:** Main characteristics of all studies of *T54A* genotypes included in the meta-analysis

							Case			Control		
First author	Year	Country or Region	Ethnicity	Case	Control	Group	AA	AG	GG	AA	AG	GG
Teng YN^20^	2002	Taiwan China	Asian	142	160	Total	121	21	0	114	2	0
Bartoloni L^21^	2004	Italy	Caucasian	71	63	Total	71	0	0	63	0	0
						OAT^a^	55	0	0			
						Azoospermia	16	0	0			
Becherini L^22^	2004	Italy	Caucasian	92	229	Total	92	0	0	229	0	0
Tschanter P^16^	2004	Germany	Caucasian	202	165	Total	202	0	0	165	0	0
Yang XJ^18^	2005	Japan	Asian	234	131	Total	234	0	0	131	0	0
						OAT^a^	93	0	0			
						Azospermia	141	0	0			
Thangaraj K^19^	2006	India	Asian	660	350	Total	660	0	0	350	0	0
						OAT^a^	62	0	0			
						Azoospermia	598	0	0			
Teng YN^23^	2006	Taiwan China	Asian	231	191	Total	205	25	1	189	2	0
Wen XH^17^	2007	Mainland China	Asian	144	53	Total	144	0	0	53	0	0
						OAT^a^	117	0	0			
Poongothai J^27^	2008	India	Asian	146	140	Total	146	0	0	140	0	0
						OAT^a^	144	0	0			
						Azoospermia	1	0	0			
Wang H^26^	2009	Mainland China	Asian	196	40	Total	192	0	4	40	0	0
Singh K^15^	2009	India	Asian	165	200	Total	165	0	0	199	1	0
Ye LW^25^	2013	Mainland China	Asian	173	175	Total	173	0	0	175	0	0
						OAT^a^	173	0	0			

OAT: oligoasthenoteratozoospermia.

**Table 3:** Main results for the *T12A* polymorphism in the meta-analysis

		AG vs. AA					GG vs. AA					AG/GG vs. AA (dominant model)					GG vs. AA/AG (recessive model)				
	Studies	OR (95% CI)	*P*	*Ph*^a^	*I^2^*	*Ph*^b^	OR (95% CI)	*P*	*Ph*^a^	*I^2^*	*Ph*^b^	OR (95% CI)	*P*	*Ph*^a^	*I^2^*	*Ph*^b^	OR (95% CI)	*P*	*Ph*^a^	*I^2^*	*Ph*^b^
**Total**	10	**1.262 (1.003–1.587)**	**0.047**	**0.991**	**0.00%**		0.868 (0.396–1.900)	0.723	0.955	0.00%		1.244 (0.994–1.557)	0.057	0.999	0.00%		0.840 (0.384–1.834)	0.661	0.957	0.00%	
***Ethnic groups***																					
**Asian**	8	1.264 (0.939–1.700)	0.122	0.984	0.00%	1.000	0.721 (0.286–1.815)	0.487	0.927	0.00%	0.476	1.232 (0.923–1.644)	0.157	0.999	0.00%	0.896	0.711 (0.283–1.789)	0.468	0.925	0.00%	0.524
**Caucasian**	2	1.259 (0.876–1.808)	0.213	0.443	0.00%	1.347 (0.323–5.619)	0.683	0.650	0.00%	1.264 (0.885–1.806)	0.198	0.508	0.00%	1.235 (0.299–5.103)	0.771	0.622	0.00%				
**Sperm concentration of case group**																					
**Azoospermia**	2	1.411 (0.934–2.129)	0.102	0.813	0.00%		0.541 (0.137–2.138)	0.381	0.210	36.40%		1.312 (0.883–1.950)	0.179	0.516	0.00%		0.523 (0.132–2.072)	0.356	0.219	33.90%	
**OAT**	5	0.986 (0.616–1.579)	0.954	0.542	0.00%		1.122 (0.265–4.763)	0.876	0.917	0.00%		0.984 (0.616–1.566)	0.946	0.644	0.00%		1.130 (0.268–4.774)	0.868	0.905	0.00%	

Test for heterogeneity ^a^in groups and ^b^between groups.

**Table 4:** Main results for the *T54A* polymorphism in the meta-analysis

		AG vs. AA					GG vs. AA					AG/GG vs. AA (dominant model)					GG vs. AA/AG (recessive model)				
	Studies	OR (95% CI)	*P*	*Ph*^a^	*I^2^*	*Ph*^b^	OR (95% CI)	*P*	*Ph*^a^	*I^2^*	*Ph*^b^	OR (95% CI)	*P*	*Ph*^a^	*I^2^*	*Ph*^b^	OR (95% CI)	*P*	*Ph*^a^	*I^2^*	*Ph*^b^
**Total**	12	**4.364 (2.207–8.630)**	**0.000**	**0.263**	**18.50%**		1.136 (0.399–3.231)	0.811	1.000	0.00%		**4.584 (2.320–9.058)**	**0.000**	**0.378**	**6.80%**		1.109 (0.390–3.157)	0.846	1.000	0.00%	
***Ethnic groups***																					
**Asian**	9	**4.842 (2.339–10.025)**	**0.000**	**0.138**	**35.00%**	0.320	1.121 (0.345–3.636)	0.850	0.998	0.00%	0.929	**5.097 (2.463–10.546)**	**0.000**	**0.231**	**23.80%**	0.291	1.087 (0.335–3.529)	0.889	0.999	0.00%	0.909
**Caucasian**	3	1.195 (0.124–11.549)	0.878	0.909	0.00%	1.195 (0.124–11.549)	0.878	0.909	0.00%	1.195 (0.124–11.549)	0.878	0.909	0.00%	1.195 (0.124–11.549)	0.878	0.909	0.00%				
**Sperm concentration of case group**																					
**Azoospermia**	6	1.922 (0.261–14.146)	0.521	0.280	21.80%		1.922 (0.261–14.146)	0.521	0.280	21.80%		1.922 (0.261–14.146)	0.521	0.280	21.80%		1.922 (0.261–14.146)	0.521	0.28	21.80%	
**OAT**	5	1.203 (0.242–5.986)	0.821	0.974	0.00%		1.203 (0.242–5.986)	0.821	0.974	0.00%		1.203 (0.242–5.986)	0.821	0.974	0.00%		1.203 (0.242–5.986)	0.821	0.974	0.00%	

Test for heterogeneity ^a^in groups and ^b^between groups.
