# Supplementary material for: Association between DAZL polymorphisms and susceptibility to male infertility: systematic review with meta-analysis and trial sequential analysis
Source: Sci Rep. 2014 Apr 10;4:4642. doi: 10.1038/srep04642 (PMC5380160; doi:10.1038/srep04642)
Supplement: Supplementary Information [file srep04642-s1.doc]

**Association between DAZL polymorphisms and susceptibility to male infertility: systematic review with meta-analysis and trial sequential analysis**

Simin Zhang ^1, 2, 3, †^, Qiuqin Tang ^4, †^, Wei Wu ^1, 2, 5, *^, Beilei Yuan ^1, 2^, Chuncheng Lu ^1, 2^, Yankai Xia ^1, 2^, Hongjuan Ding ^4^, Lingqing Hu ^5^, Daozhen Chen ^5^, Jiahao Sha ^1^, Xinru Wang ^1, 2^

Table S1. Appraisal of quality (Newcastle-Ottawa Scale) of included studies.

| **Study** | | **Selection** | | | | **Comparability** | | **Exposure** | | | **Score** |
| --- | --- | --- | --- | --- | --- | --- | --- | --- | --- | --- | --- |
|  |  | Adequate case definition | Case representative | Control selection | Control definition | Comparability by design | Comparability by analysis | Same ascertainment for cases and controls | | Nonresponse rate reported |  |
|  |  |  |  |  |  |  |  | Method | Same |  |  |
| Teng YN | 2002 | 1 | 1 | 1 | 1 | 0 | 1 | 1 | 1 | 1 | 8 |
| Bartoloni L | 2004 | 1 | 1 | 0 | 1 | 0 | 1 | 0 | 1 | 1 | 6 |
| Becherini L | 2004 | 1 | 1 | 1 | 1 | 0 | 1 | 1 | 1 | 1 | 8 |
| Tschanter P | 2004 | 1 | 1 | 0 | 1 | 0 | 1 | 1 | 1 | 1 | 7 |
| Yang XJ | 2005 | 1 | 1 | 0 | 1 | 1 | 1 | 0 | 1 | 1 | 7 |
| Thangaraj K | 2006 | 1 | 1 | 1 | 1 | 0 | 1 | 1 | 1 | 1 | 8 |
| Teng YN | 2006 | 1 | 1 | 1 | 1 | 0 | 1 | 1 | 1 | 1 | 8 |
| Wen XH | 2007 | 1 | 0 | 0 | 1 | 1 | 1 | 0 | 1 | 1 | 6 |
| Poongothai J | 2008 | 1 | 0 | 0 | 1 | 0 | 1 | 1 | 1 | 1 | 6 |
| Wang H | 2009 | 1 | 1 | 0 | 1 | 0 | 1 | 1 | 1 | 1 | 7 |
| Singh K | 2009 | 1 | 0 | 1 | 1 | 1 | 1 | 0 | 1 | 1 | 7 |
| Kumar K | 2011 | 1 | 1 | 1 | 1 | 1 | 1 | 0 | 1 | 1 | 8 |
| Ye LW | 2013 | 1 | 1 | 0 | 1 | 1 | 1 | 1 | 1 | 1 | 8 |

Notes: The studies are judged on three broad perspectives using a nine point scale according to the Newcastle-Ottawa quality assessment scale: selection of the study group (0-4 points), comparability of cohorts (0-2 points) and ascertainment of outcome (0-3 points).

**Figure S1. Forest plot of the *T12A* polymorphism and male infertility risk in the dominant model and recessive model.** Studies are plotted according to the last name of the first author and followed by the publication year in parentheses. Horizontal lines represent 95% CI. Each square represents the OR point estimate and its size is proportional to the weight of the study. The diamond (and broken line) represents the overall summary estimate, with confidence interval given by its width. The unbroken vertical line is at the null value (OR = 1.0). CI, confidence interval; OR, odds ratio.


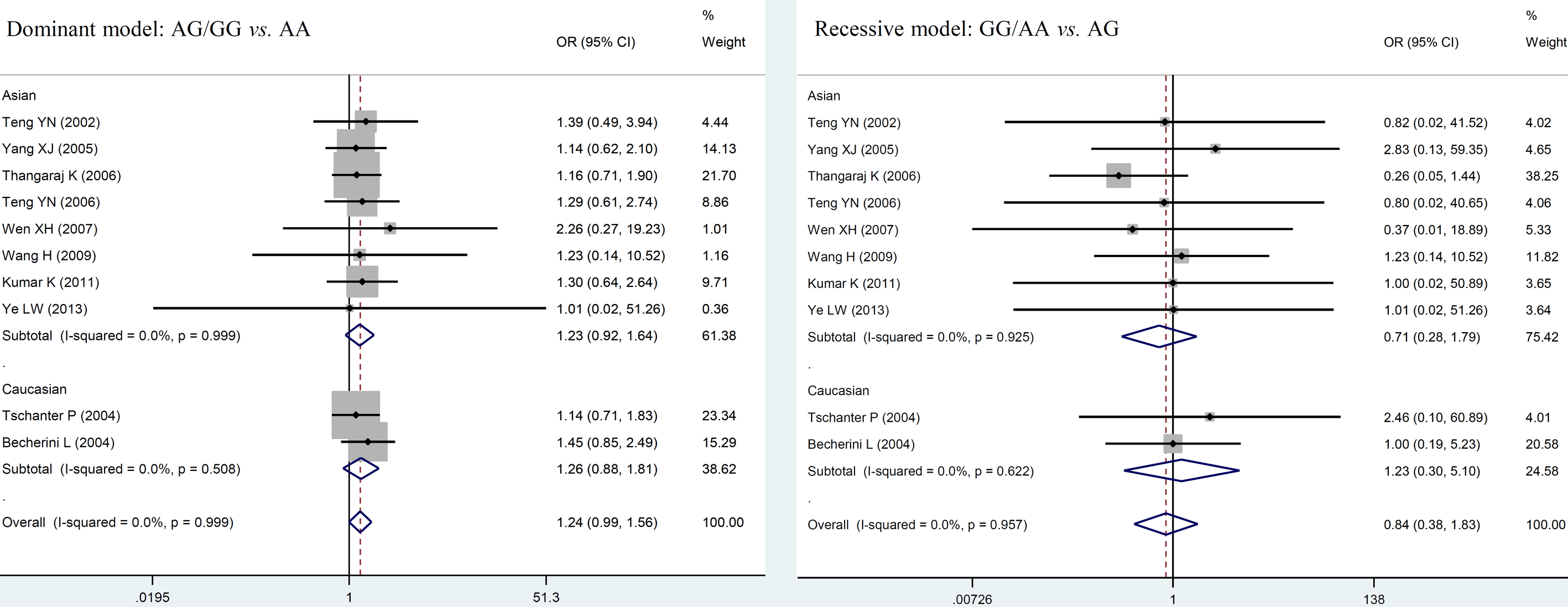


**Figure S2. Funnel plot for *T12A* analysis to detect publication bias.** Each dot represents a separate study for the indicated association. Location outside the delineated triangle (pseudo 95%CI) suggests a publication bias.


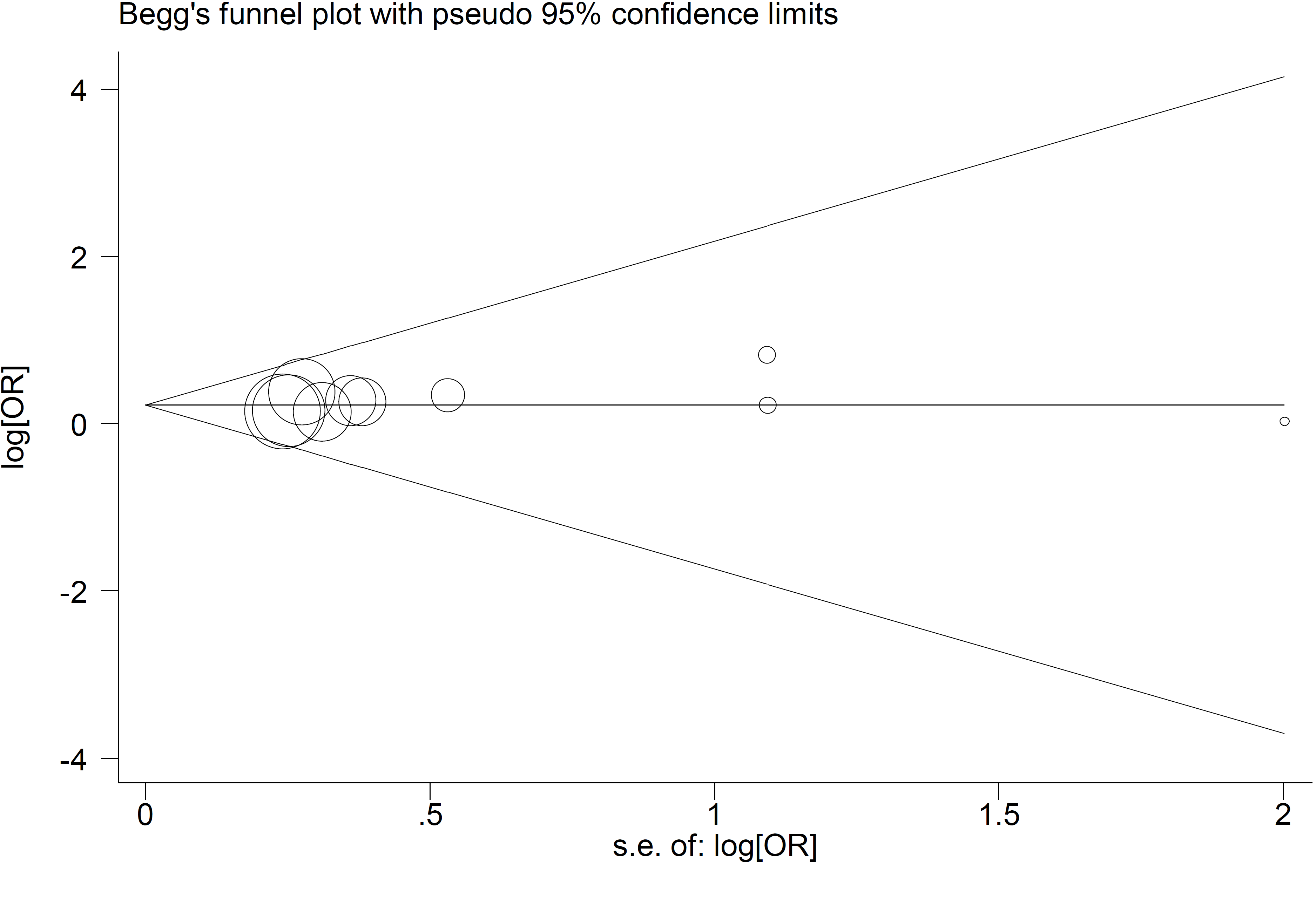


**Figure S3. Sensitivity of *T12A* and *T54A* individual studies used in the meta-analysis under co-dominant model.** The horizontal axis represents the odds ratio. Every circle indicates the pooled OR when the left study is omitted in this meta-analysis. The two ends of every broken line represent the respective 95% CI.

**
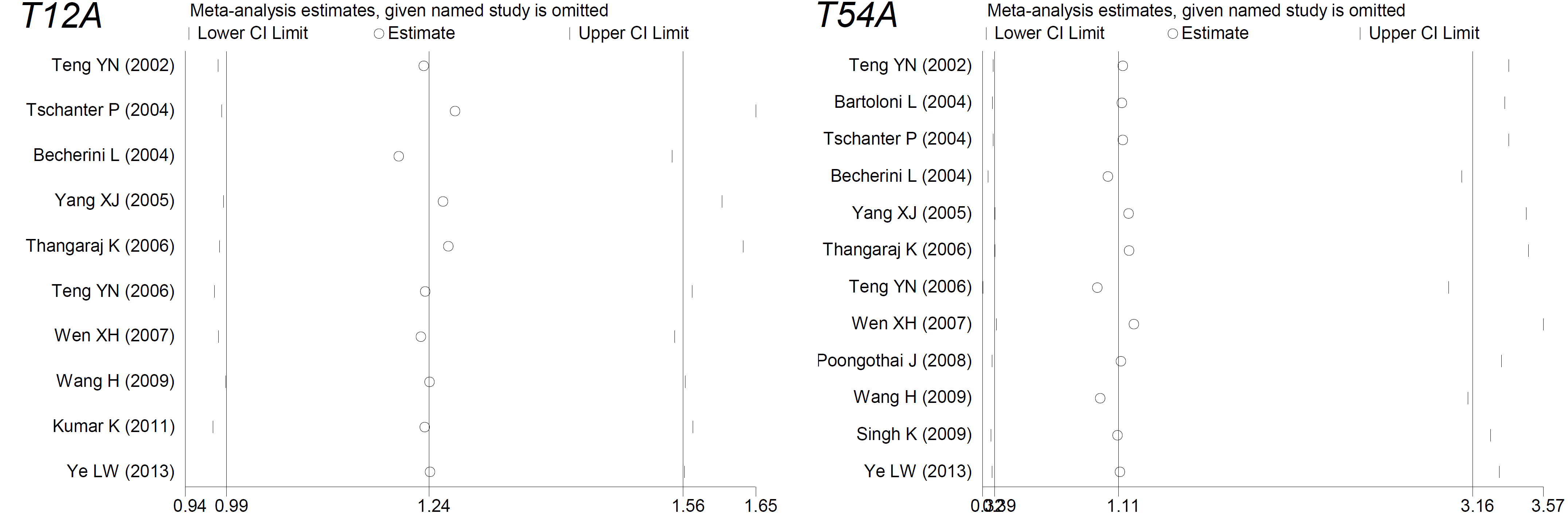
**
